# Postoperative Outcomes and Functional Recovery After Preoperative Combination Chemotherapy for Pancreatic Cancer: A Propensity Score-Matched Study

**DOI:** 10.3389/fonc.2019.01299

**Published:** 2019-11-26

**Authors:** Nicolò Pecorelli, Michele Pagnanelli, Lorenzo Cinelli, Francesca Di Salvo, Stefano Partelli, Stefano Crippa, Domenico Tamburrino, Renato Castoldi, Giulio Belfiori, Michele Reni, Massimo Falconi, Gianpaolo Balzano

**Affiliations:** ^1^Division of Pancreatic Surgery, Pancreas Translational & Clinical Research Center, San Raffaele Scientific Institute, Milan, Italy; ^2^Vita-Salute San Raffaele University, Milan, Italy; ^3^Department of Medical Oncology, San Raffaele Scientific Institute, Milan, Italy

**Keywords:** pancreatic cancer, neoadjuvant treatment, pancreaticoduodenectomy, postoperative complications, functional recovery

## Abstract

**Introduction:** Previous studies show encouraging oncologic outcomes for neoadjuvant chemotherapy (NACT) in the setting of pancreatic ductal adenocarcinoma (PDAC). However, recent literature reported an increased clinical burden in patients undergoing pancreaticoduodenectomy (PD) following NACT. Therefore, the aim of our study was to assess the impact of NACT on postoperative outcomes and recovery after PD.

**Methods:** A retrospective propensity score-matched study was performed including all patients who underwent PD for PDAC in a single center between 2015 and 2018. Patients treated with NACT for resectable, borderline resectable or locally advanced PDAC were matched based on nearest neighbor propensity scores in a 1:1 ratio to patients who underwent upfront resection. Propensity scores were calculated using 7 perioperative variables, including gender, age, BMI, ASA score, Charlson-Deyo comorbidity score, fistula risk score (FRS), vascular resection. Primary outcome was the number and severity of complications at 90-days after surgery measured by the comprehensive complication index (CCI). Data are reported as median (IQR) or number of patients (%).

**Results:** Of 283 resected patients, 95 (34%) were treated with NACT. Before matching, NACT patients were younger, had less comorbidities (Charlson-Deyo score 0 vs. 1, *p* = 0.04), similar FRS [2 (0–3) for both groups], and more vascular resections performed [*n* = 28 (30%) vs. *n* = 26 (14%), *p* < 0.01]. After propensity-score matching, preoperative and intraoperative characteristics were comparable. Postoperatively, CCI was similar between groups [8.7 (0–29.6) for both groups, *p* = 0.59]. NACT patients had a non-statistically significant increase in superficial incisional surgical site infections [*n* = 12 (13%) vs. 6 (6%), *p* = 0.14], while no difference was found for overall infectious complications and organ-space SSI. The occurrence of clinically-relevant pancreatic fistula was similar between groups [10 (11%) vs. 13 (14%), *p* = 0.51]. No difference was found between groups for length of hospital stay [8 (7–15) vs. 8 (7–14) days, *p* = 0.62], and functional recovery outcomes.

**Conclusion:** After propensity score adjustment for perioperative risk factors, NACT did not worsen postoperative outcomes and functional recovery following PD for PDAC compared to upfront resection.

## Introduction

Preoperative oncological therapy is widely used in the treatment of localized pancreatic adenocarcinoma. At present, it is mainly offered to patients with borderline or locally advanced tumors, but it is increasingly administered to patients with resectable cancer (1, 2). In fact, neoadjuvant chemotherapy (NACT) provides several theoretical advantages compared to adjuvant therapy even for resectable tumors; it allows early treatment of micro-metastases, increases R0/N0 resection rate, enables a better drug diffusion into tissues, and allows to treat a greater number of patients, that could be prevented from adjuvant treatment because of surgical complications or poor surgical recovery (3). A recent randomized phase II trial showed a significant survival improvement in resectable cancer by the perioperative administration of an effective combination chemotherapy (4).

A widespread concern in applying preoperative therapy is an increase of postoperative complications after pancreatic resection, already burdened with high morbidity and mortality rates (5, 6). Several factors could potentially affect postoperative outcome: (i) an impairment of liver and bone marrow function; (ii) a worsening of nutritional status and sarcopenia, which is known to impact on surgical outcomes (7); (iii) the detrimental effect of a biliary stent placement in jaundiced patients, causing an increased morbidity in patients potentially candidate to upfront surgery (8). Despite these risks, most published studies do not indicate an increase of complications in patients treated with neoadjuvant therapy, when compared to those undergoing upfront surgery (9–11). However, most studies are retrospective, with a small sample size and carry a great variability in the administered therapy, preventing us from concluding that preoperative treatment does not affect surgical outcome. Further, more complex combination chemotherapy regimens, mainly FOLFIRINOX or gemcitabine plus nab-paclitaxel, have been introduced in recent years and have become the standard for pancreatic cancer treatment. To decide whether a patient with borderline resectable or resectable pancreatic cancer should be a candidate to neoadjuvant treatment, a crucial information regards the possibility that preoperative therapy could worsen the surgical outcome. Postoperative complications affect postoperative recovery and hospitalization costs, and could lead to reduced overall survival, compromising the potential advantage offered by preoperative treatment (12).

The objective of the present study was to assess the extent to which preoperative chemotherapy impacts on postoperative morbidity and functional recovery in patients undergoing pancreaticoduodenectomy (PD) at our Institution in a recent time frame. The findings of the present study should contribute evidence toward the effect of current preoperative combination chemotherapy on operative outcome.

## Methods

This propensity-score matched retrospective study was conducted at San Raffaele Hospital, a high-volume referral center for pancreatic surgery (13). All patients scheduled for elective PD for PDAC between January 2015 and September 2018 were considered eligible for the study. Patients who successfully underwent planned resection were included in the study. Before surgery, all patients signed an informed consent for the planned procedure. The need for ethical approval was waived by the San Raffaele Hospital ethical review board due to the retrospective nature of the study.

### Indication to Upfront Surgery vs. Neoadjuvant Chemotherapy

Patients with a pathological diagnosis of PDAC were evaluated at diagnosis by an attending surgeon at our institution and discussed at a multidisciplinary meeting including a medical oncologist expert in pancreatic cancer treatment and an experienced radiologist. Indication to upfront surgery vs. neoadjuvant chemotherapy was based upon review of radiological imaging including a three-phase, high-resolution chest and abdomen contrast-enhanced CT scan performed within 4 weeks, biochemical testing including carcinoma antigen (CA) 19-9 and carcinoembryonic antigen (CEA).

Indication to neoadjuvant treatment was mainly given for borderline resectable and locally advanced disease defined on the basis of the National Comprehensive Cancer Network (NCCN). Patients were also considered for neoadjuvant chemotherapy in case of biological or clinical factors suspicious for metastatic disease such as significantly increased CA19-9, chronic pain or other conditional factors as a low performance status, as suggested by the 2017 International consensus on definition and criteria of borderline resectable pancreatic ductal adenocarcinoma (14). In a minority of cases, neoadjuvant chemotherapy was also proposed to patients with resectable disease.

Neoadjuvant chemotherapy was administered at our institution or at another cancer center closer to the patient's home. Chemotherapy regimen and dosage was chosen by the treating oncologist. Most commonly patients received fluorouracil + leucovorin + irinotecan + oxaliplatin (mFOLFIRINOX) (15), gemcitabine + nab-paclitaxel (16), or other gemcitabine-based combination chemotherapy. Restaging CT scan was usually performed at 2–3 months after the start of chemotherapy based on institutional protocols and repeated at the same interval until the end of chemotherapy. Chemotherapy lasted a minimum of 6 cycles (14-days per cycle) for mFOLFIRINOX and 4 cycles for gemcitabine-based regimens (28-days per cycle). At every radiological assessment, patients were evaluated for resectability by the multidisciplinary team. Surgery was indicated when a gross radical resection could be predicted in absence of radiological or biological (CA 19-9) tumor progression. A small number of patients was also treated with preoperative radiotherapy, as a result of multidisciplinary team discussion.

### Perioperative Management

Laparoscopic exploration was only performed in patients with a high clinical suspicion for metastatic disease. Determinants of intraoperative resectability were absence of distant metastases, reconstructible superior mesenteric vein/portal vein, and no need for superior mesenteric artery resection. A pylorus-preserving pancreaticoduodenectomy with standard lymphadenectomy was the routinely performed procedure. A two-layer end-to-side pancreatico-jejunostomy, a single-layer interrupted suture end-to-side hepatico-jejunostomy, and a single-layer interrupted suture end-to-side duodeno-jejunostomy were carried out on the same jejunal loop. Two drains were usually placed close to biliary and pancreatic anastomosis. All patients were managed according to an enhanced recovery after surgery protocol and discharged after meeting predefined criteria, as previously described (17, 18).

### Data Collection

Data was retrieved from our institutional electronic pancreatic surgery database. Before surgery, demographics, comorbidities, American Society of Anesthesiologists (ASA) score, body mass index (BMI), Charlson-Deyo comorbidity score (19), serum hemoglobin were recorded in all patients. Duration of surgery, operative blood loss, surgeon assessment of main pancreatic duct diameter and pancreatic stump texture were also recorded. The fistula risk score (FRS) was calculated according to Callery et al. (20). Briefly, the FRS is a score ranging from 0 to 10 based on main pancreatic duct diameter, pancreatic texture, pancreatic underlying disease and intraoperative blood loss amount. In this study, involving only patients with PDAC, the FRS highest possible value would be 9.

Postoperative 90-day office and telephone follow-up was routinely carried out to record morbidity, mortality and readmissions.

### Outcome Measures

Primary outcome of the study was the comprehensive complication index (CCI) at 90-days after surgery. This is a validated measure summarizing the number of complications occurred and their severity in a single score ranging from 0 to 100 based on the Dindo-Clavien classification (21, 22). Zero corresponds to no postoperative complication, 100 to mortality.

Secondary outcomes included patient functional recovery measures, specific postoperative complications and length of hospital stay (LOS). Functional recovery was assessed evaluating time to first oral liquids and solid intake, time to recovery of gastrointestinal function, time to suspension of intravenous fluids and removal of urinary catheter. Clinically relevant pancreatic fistula was considered as grade B or C according to ISGPS criteria (23). Surgical site infections (SSI) were classified as superficial incisional, deep incisional or organ-space according to the definition by the Center for Disease Control (CDC) (24). Microbiological analysis and positive culture proved all infectious complications. Post-pancreatectomy hemorrhage (PPH) and delayed gastric emptying (DGE) were defined according to ISGPS definitions (25, 26).

### Statistical Analysis

In order to reduce selection bias, a propensity score matched cohort analysis was performed to evaluate the extent to which neoadjuvant chemotherapy was associated with an increase in postoperative morbidity. Propensity scores for all patients were calculated using a logistic regression model based on the following preoperative and intraoperative prognostic factors: age, sex, BMI, Fistula Risk Score, ASA score, Charlson-Deyo comorbidity score and vascular resection. Once propensity score was derived, a neighbor-matching algorithm was used to match patients who received neoadjuvant treatment with those who underwent upfront surgery.

Descriptive data are reported as mean (standard deviation), or median (interquartile range, IQR), otherwise specified. Comparisons between groups were evaluated by using Fisher's exact test and Chi-square test for categorical variables, or two-tailed Student's *t*-test or Mann-Whitney U test for continuous variables, as appropriate. Statistical analysis was performed using the *MatchIt* R package (version 3.0.3, R Foundation for Statistical Computing— www.r-project.org/) and STATA® version 13.1 software (StataCorp, College Station, TX, USA). All statistical tests were 2-sided, a “*p*” < 0.05 was considered to indicate statistical significance.

## Results

Overall, 327 patients underwent elective surgical exploration for a planned PD for PDAC during the study period. At surgery, 44 (13%) patients were not resected because of liver metastasis (*n* = 21, 6%), peritoneal carcinomatosis (*n* = 7, 2%), positive paraaortic lymph-nodes (*n* = 4, 1%), or locally unresectable disease (*n* = 12, 4%). Therefore, 283 patients who finally underwent PD were included in this study. Of these, 188 had an upfront resection (66%) while 95 had previously received neoadjuvant chemotherapy (34%).

Table 1 includes the characteristics of neoadjuvant treatment in 95 patients who received preoperative chemotherapy included in the study. The most common regimens used were Gemcitabine + Nab-Paclitaxel (*n* = 44, 46%) or mFOLFIRINOX (*n* = 35, 37%). Median duration of chemotherapy was 4 (IQR 4–6) months.

**Table 1:** Characteristics of neoadjuvant therapy in 95 treated patients.

**Variables**	***n =* 95**
Indication to preoperative chemotherapy	
Anatomic borderline resectable disease	45 (47.4%)
Locally advanced disease	26 (27.4%)
Biologic borderline resectable disease	14 (23.2%)
Resectable disease	8 (8.4%)
Metastatic disease	2 (2.1%)
Chemotherapeutic regimen	
mFOLFIRINOX	35 (36.8%)
Gemcitabine + Nab-Paclitaxel	44 (46.3%)
Gemox	9 (9.5%)
PAX-G	7 (7.4%)
Duration of chemotherapy (months)^a^	4 (4–6)
Less than 6 months	68 (71.6%)
6 or more months	27 (28.4%)
Associated preoperative radiotherapy	12 (12.6%)

*Values are number of patients (%), or ^a^median (interquartile range). mFOLFIRINOX: fluorouracil, leucovorin, irinotecan, oxaliplatin; Gemox: gemcitabine, oxaliplatin; PAX-G: cisplatin, nab-paclitaxel, oral capecitabine, and gemcitabine*.

Table 2 reports preoperative and intraoperative characteristics of the entire cohort. Before matching, patients in the upfront resection group were older (*n* = 90, 48% vs. *n* = 31, 33% were 70-years or older; *p* = 0.01) and had more comorbidities with an increased Charlson-Deyo comorbidity index (median 1 vs. 0; *p* = 0.04), a higher incidence of coronary artery disease [*n* = 29 (15%) vs. 1 (1%); *p* < 0.01] and COPD [*n* = 15 (8%) vs. 1 (1%); *p* = 0.02]. During surgery, more vascular resections were performed in NACT (*n* = 28, 30%) vs. upfront resection (*n* = 26, 14%) patients (*p* < 0.01). No difference was found between groups for FRS and characteristics of the pancreatic stump. After propensity score adjustment (Table 3), the two groups appeared balanced for preoperative and surgical characteristics, with the only significant differences between groups in coronary artery disease and COPD incidence.

**Table 2:** Characteristics of the entire cohort.

**Variables**	**Overall *n =* 283**	**Neoadjuvant *n =* 95**	**Upfront resection *n =* 188**	***p*-value**
Preoperative variables				
Age^a^	68 (62–74)	67 (60–72)	69 (63–75)	<0.01
Elderly (70+ years	121 (42.8%)	31 (32.6%)	90 (47.9%)	0.01
old)				
Male gender	146 (51.6%)	45 (47.4%)	101 (53.7%)	0.31
BMI (kg/m^2^)^b^	24.3 (0.4)	24.2 (0.4)	24.4 (0.3)	0.73
Overweight (BMI ≥	100 (35.3%)	30 (31.6%)	70 (37.2%)	0.35
25 kg/m^2^)				
ASA score 3+	117 (41.3%)	43 (45.3%)	74 (39.4%)	0.34
Charlson	1 (0–1)	0 (0–1)	1 (0–1)	0.04
Comorbidity index^a^				
Pre-existing comorbidities				
Coronary artery disease	30 (10.6%)	1 (1.1%)	29 (15.4%)	<0.01
COPD	16 (5.6%)	1 (1.1%)	15 (8.0%)	0.02
Diabetes	86 (30.4%)	26 (27.4%)	60 (31.9%)	0.43
Chronic kidney disease	11 (3.9%)	2 (2.1%)	9 (4.8%)	0.35
Preoperative biliary drainage	182 (64.4%)	62 (65.3%)	120 (63.8%)	0.74
Preoperative hemoglobin (g/dL)^b^	12.5 (0.2)	12.5 (0.1)	12.5 (0.1)	0.73
Surgical variables				
Operative time (minutes)^a^	315 (280–351)	336 (291–385)	305 (278–334)	<0.01
Operative blood loss (mL)^a^	300 (200–450)	350 (200–500)	300 (200–405)	0.34
Fistula risk score^a^	2 (0–3)	2 (0–3)	2 (0–3)	0.38
>2	97 (34.3%)	27 (28.4%)	70 (37.2%)	0.14
Soft pancreas	99 (35%)	29 (30.5%)	70 (37.2%)	0.26
Vascular resection	54 (19.1%)	28 (29.5%)	26 (13.8%)	<0.01

*Data are number of patients (%), or ^a^median (interquartile range), or ^b^mean (standard deviation)*.

*BMI, Body Mass Index; ASA, American Society of Anesthesiologists; COPD, chronic obstructive pulmonary disease*.

**Table 3:** Preoperative and surgical characteristics after propensity score adjustment.

**Variables**	**Overall *n =* 190**	**Neoadjuvant *n =* 95**	**Upfront resection *n =* 95**	***p*-value**
Preoperative variables				
Age^a^	66 (59–73)	67 (60–72)	66 (59–73)	0.60
Elderly (70+ years	65 (34.2%)	31 (32.6%)	34 (35.8%)	0.65
old)				
Male gender	92 (48.4%)	45 (47.4%)	47 (49.5%)	0.77
BMI (kg/m^2^)^b^	24.1 (0.2)	24.2 (0.4)	24.0 (0.3)	0.77
Overweight (BMI ≥	62 (32.6%)	30 (31.6%)	32 (33.7%)	0.75
25 kg/m^2^)				
ASA score 3+	86 (45.3%)	43 (45.3%)	43 (45.3%)	0.99
Charlson	0 (0–1)	0 (0–1)	0 (0–1)	0.96
Comorbidity index^a^				
Pre-existing comorbidities				
Coronary artery disease	12 (6.3%)	1 (1.1%)	11 (11.6%)	<0.01
COPD	8 (4.2%)	1 (1.1%)	7 (7.4%)	0.03
Diabetes	49 (25.8%)	26 (27.4%)	23 (24.2%)	0.61
Chronic kidney disease	4 (2.1%)	2 (2.1%)	2 (2.1%)	0.99
Preoperative biliary drainage	121 (63.7%)	62 (65.3%)	59 (62.1%)	0.65
Preoperative hemoglobin (g/dL)^b^	12.5 (0.1)	12.5 (0.1)	12.4 (0.2)	0.77
Surgical variables				
Operative time (minutes)^a^	317.5 (287–360)	336 (291–385)	307 (280–340)	<0.01
Operative blood loss (mL)^a^	300 (200–450)	350 (200–500)	300 (200–400)	0.11
Fistula risk score^a^	2 (0–3)	2 (0–3)	2 (0–3)	0.33
>2	54 (28.4%)	27 (28.4%)	27 (28.4%)	0.99
Soft pancreas	57 (30%)	29 (30.5%)	28 (29.5%)	0.87
Vascular resection	49 (25.8%)	28 (29.5%)	21 (22.1%)	0.24

*Data are number of patients (%), or ^a^median (interquartile range), or ^b^mean (standard deviation)*.

*BMI, Body Mass Index; ASA, American Society of Anesthesiologists; COPD, chronic obstructive pulmonary disease*.

Table 4 shows postoperative functional recovery outcomes in the two groups. No difference was found between groups for time to recovery of oral intake, recovery of gastrointestinal function and other functional recovery variables.

**Table 4:** Postoperative functional recovery outcomes.

**Variables**	**Overall *n =* 190**	**Neoadjuvant *n =* 95**	**Upfront resection *n =* 95**	***p*-value**
Time to first oral liquids	1 (1–1)	1 (1–2)	1 (1–2)	0.87
Time to first solid food	2 (2–3)	2 (2–3)	2 (2–3)	0.28
Time to recovery of gastrointestinal function	4 (3–4)	4 (3–4)	4 (3–4)	0.87
Time to intravenous fluid infusion stop	3 (2–5)	3 (2–5)	3 (2–5)	0.51
Time to urinary catheter removal	2 (2–3)	2 (2–3)	2 (2–3)	0.15

*Data are median (interquartile range) postoperative days*.

Postoperative outcomes are reported in Table 5. For the primary outcome measure of the study, the CCI, there was no difference between groups with a median value of 8.7 (0–29.6) in both groups. This corresponds to a single Dindo-Clavien grade 1 complication in both groups. Major complications occurred in 20 (21%) patients of the NACT and 15 (16%) of the upfront resection group (*p* = 0.35). Mortality at 90-days occurred in 1 patient (1%) per group. Both deaths were due to multiple organ failure after a complicated postoperative course including relaparotomy for late occurrence of duodeno-jejunal anastomotic leak. Need for surgical reintervention was similar between groups, *n* = 8 (8%) in the NACT and *n* = 3 (3%) in the upfront resection (*p* = 0.21). Causes of reoperation in the NACT group were duodenojejunal anastomotic leak (*n* = 2), wound complications (*n* = 2), suspected intestinal ischemia (*n* = 2), hepatico-jejunostomy leakage (*n* = 1), intra-abdominal bleeding (*n* = 1). In the upfront resection group relaparotomy was performed because of postoperative bleeding (*n* = 2) and duodenojejunal anastomotic leak (*n* = 1). Clinically relevant pancreatic fistula occurred in 23 patients (12%) with no significant difference between groups. No difference was found for overall incidence of SSI, 23% in the NACT group vs. 18% in the upfront resected, but there was a trend for increased superficial incisional SSI in the NACT group [*n* = 12 (13%)] compared to the upfront resection group [*n* = 6 (6%)] (*p* = 0.14). Median LOS was 8-days in both groups with no difference in hospital readmissions [*n* = 12 (13%) in the NACT groups vs. *n* = 9 (10%) in the upfront resection group, *p* = 0.48]. Perioperatively, the need for blood transfusion was similar between groups [NACT *n* = 25 (26.3%) vs. upfront resection *n* = 22 (23.2%); *p* = 0.62].

**Table 5:** Postoperative outcomes.

**Variables**	**Overall *n =* 190**	**Neoadjuvant *n =* 95**	**Upfront resection *n =* 95**	***p*-value**
90-day CCI^a^	8.7 (0-29.6)	8.7 (0-29.6)	8.7 (0-29.6)	0.58
Any 90-day complication	97 (51.1%)	49 (51.6%)	48 (50.5%)	0.88
90-day complication severity				
Minor Complications (Grade I–II)	62 (32.6%)	29 (30.5%)	33 (34.7%)	0.54
Major Complications (Grade III–V)	35 (18.4%)	20 (21.1%)	15 (15.8%)	0.35
90-day mortality	2 (1.1%)	1 (1.1%)	1 (1.1%)	1.00
90-day surgical reintervention	11 (5.8%)	8 (8.4%)	3 (3.2%)	0.21
Clinically-relevant pancreatic fistula	23 (12.1%)	10 (10.5%)	13 (13.7%)	0.51
Infectious complication	56 (29.5%)	30 (31.6%)	26 (27.4%)	0.52
Surgical Site Infection	39 (20.5%)	22 (23.2%)	17 (17.9%)	0.37
Organ-space SSI	29 (15.3%)	14 (14.7%)	15 (15.8%)	0.84
Superficial incisional SSI	18 (9.5%)	12 (12.6%)	6 (6.3%)	0.14
Post-pancreatectomy hemorrhage	8 (4.2%)	5 (5.3%)	3 (3.3%)	0.72
Bile leakage	15 (7.9%)	9 (9.5%)	6 (6.3%)	0.42
Delayed Gastric Emptying	31 (16.3%)	14 (14.7%)	17 (17.9%)	0.56
Cardio-respiratory complications	13 (6.8%)	9 (9.5%)	4 (4.2%)	0.15
Acute kidney injury	3 (1.6%)	1 (1.1%)	2 (2.1%)	0.56
PONV	51 (26.8%)	29 (30.6%)	22 (23.2%)	0.25
Blood transfusion^^*^^	47 (24.7%)	25 (26.3%)	22 (23.2%)	0.62
Length of hospital stay (days)^a^	8 (7-14)	8 (7-15)	8 (7-14)	0.61
90-day hospital readmission	21 (11.5%)	12 (12.6%)	9 (9.5%)	0.48
90-day post-discharge ER visits	25 (13.2%)	14 (14.7%)	11 (11.6%)	0.52

*Data are number of patients (%), or ^a^median (interquartile range)*.

* *Refers to both intra and postoperative transfusions*. *CCI, comprehensive complication index; SSI, Surgical site infection; PONV, postoperative nausea and vomit; ER, emergency room*.

Pathological findings are shown in Table 6. Patients treated with neoadjuvant chemotherapy had a significantly lower pathological stage compared to upfront resected group (*p* < 0.01) due to an increased rate of node-negative disease (34% in the NACT vs. 13% in the upfront resection group; *p* < 0.01). In the NACT group, patients presented with a more differentiated tumor compared to upfront resected patients (*p* = 0.03).

**Table 6:** Pathological findings after propensity score adjustment.

**Variables**	**Overall *n =* 190**	**Neoadjuvant *n =* 95**	**Upfront resection *n =* 95**	***p*-value**
TNM stage^a^				
1a	22 (11.6%)	14 (14.7%)	8 (8.4%)	<0.01
1b	21 (11.1%)	17 (17.9%)	4 (4.2%)	
2a	0 (0%)	0 (0%)	0 (0%)	
2b	84 (44.2%)	45 (47.4%)	39 (41.1%)	
3	60 (31.6%)	16 (16.8%)	44 (46.3%)	
4	3 (1.6%)	3 (3.2%)	0 (0%)	
Pathological *N* stage				
pN0	44 (23.2%)	32 (33.7%)	12 (12.6%)	<0.01
pN1	85 (44.7%)	46 (48.4%)	39 (41.1%)	
pN2	61 (32.1%)	17 (17.9%)	44 (46.3%)	
Lymph-nodes				
Collected	26 (19-32)	26 (19-31)	26 (19-33)	0.40
Positive	2 (1-5)	1 (0-3)	3 (1-7)	<0.01
Node ratio (%)	7.3 (2.4–18.8)	4.5 (0–12)	12.5 (4.3–23.8)	<0.01
Tumor Grading				
1–2	42 (44.2%)	57 (60%)	99 (52.1%)	0.03
3	53 (55.8%)	38 (40%)	91 (47.9%)	

*Values are number of patients (%) or ^a^median (IQR)*.

a *TNM AJCC stage 8th edition*.

A subgroup analysis in patients undergoing NACT (*n* = 95) was performed to assess possible differences in patients treated with gemcitabine-based combination chemotherapy (*n* = 65) vs. mFolfirinox regimen (*n* = 30). No significant differences were found between groups regarding CCI or other specific complications (Table 7).

**Table 7:** Postoperative outcomes in neoadjuvant group stratified by chemotherapy regimen.

**Variables**	**Overall *n =* 95**	**Gemcitabine-based *n =* 60**	**mFOLFIRINOX *n =* 35**	***p*-value**
90-day CCI^a^	8.7 (0–29.6)	8.7 (0–30.2)	8.7 (0.29.6)	0.93
Any 90-day complication	49 (51.6%)	31 (51.7%)	18 (51.4%)	0.98
90-day complication severity				
Minor Complications (Grade I–II)	29 (30.5%)	18 (30%)	11 (31.4%)	0.88
Major Complications (Grade III–V)	20 (21.1%)	13 (22%)	7 (20%)	0.85
90-day mortality	1 (1.1%)	0	1 (2.9%)	0.19
90-day surgical reintervention	8 (8.4%)	4 (6.7%)	4 (11.4%)	0.42
Clinically-relevant pancreatic fistula	10 (10.5%)	7 (11.7%)	3 (8.6%)	0.63
Infectious complication	30 (31.6%)	21 (35%)	9 (25.7%)	0.35
Surgical Site Infection	22 (23.2%)	15 (25.0%)	7 (20%)	0.58
Organ-space SSI	14 (14.7%)	10 (16.7%)	4 (11.4%)	0.49
Superficial incisional SSI	12 (12.6%)	7 (11.7%)	5 (14.3%)	0.71
Post-pancreatectomy hemorrhage	5 (5.3%)	3 (5%)	2 (5.7%)	0.89
Bile leakage	9 (9.5%)	5 (8.3%)	4 (11.4%)	0.72
Delayed Gastric Emptying	14 (14.7%)	9 (15%)	5 (14.3%)	0.92
Cardio-respiratory complications	9 (9.5%)	7 (11.7%)	2 (5.7%)	0.34
Length of hospital stay (days)^a^	8 (7-15)	9 (7-15)	8 (6-18)	0.36
90-day hospital readmission	12 (12.6%)	7 (11.7%)	5 (14.3%)	0.71
90-day post-discharge ER visits	14 (14.7%)	8 (13.3%)	6 (17.4%)	0.61

*Data are number of patients (%), or ^a^median (interquartile range)*.

*CCI, comprehensive complication index; SSI, Surgical site infection; ER, emergency room*.

## Discussion

The adoption of preoperative chemotherapy for non-metastatic pancreatic cancer has increased significantly in recent years with the advent of more effective drug combination chemotherapy regimens, but there is still limited evidence on the effect of such treatment of surgical outcomes after pancreatectomy. In this propensity-score matched retrospective study performed at a high-volume referral center for pancreatic surgery, we found that preoperative combination chemotherapy did not increase postoperative morbidity nor worsen functional recovery. Our analysis was limited to a recent 3-year period, providing an updated picture of the impact of preoperative chemotherapy with postoperative outcomes in the era of drug combination chemotherapy.

In pancreatic cancer treatment, preoperative chemotherapy is now routinely offered to patients with locally advanced or borderline resectable disease. However, biological factors such as a high tumor marker at diagnosis, which correlates with presence of micrometastatic disease and worse prognosis, or a low patient performance status may also shift the indication from upfront resection to neoadjuvant treatment (14). Interestingly, in our cohort, before propensity score adjustment, patients in the upfront resection group were older and had more concomitant morbidity. After matching, the two groups were similar, but a higher incidence of coronary artery disease and COPD was still present in the group of patients treated with upfront resection. It is possible that these comorbidities also influenced the decision to proceed with resection as the first therapeutic approach, fearing that comorbidities could affect CT tolerance and effectiveness.

Most surgeons roughly consider preoperative therapy as a uniform treatment, whereas there is a set of different therapies that have been applied to date, including single agent chemotherapy or combination chemotherapy, with short (<3 months) or long duration (4–6 months or even longer); or chemoradiotherapy with different doses, different fractions and different methods. A short course chemotherapy with single agent gemcitabine, widely used in the past, could differently affect the operative outcome with respect to a long-term combination chemotherapy, that is currently offered to patients with pancreatic cancer. In the present study, all patients received combination regimens with more than 90% receiving mFOLFIRINOX or gemcitabine plus nab-paclitaxel, with a minimum treatment duration of 4 months.

The comparison between the two groups confirmed that neoadjuvant combination chemotherapy did not significantly affect the perioperative course: no increase in complications was recorded (assessed both in terms of CCI, and by traditional postoperative outcomes), and no difference in perioperative blood transfusions. The CCI is a recently developed and validated metric accounting for both the number and severity of complications. It has been advocated as a more sensitive endpoint for assessing differences in morbidity compared to traditional measures (27). In our analysis CCI was equivalent in both groups. All patients were treated with the same ERAS protocol (17, 18) including early oral feeding, mobilization out of bed, and removal of urinary catheter. The adherence to enhanced recovery elements was not different in the two groups leading to similar time to postoperative functional recovery, length of hospital stay and readmissions after discharge. Patients treated with preoperative chemotherapy were expected to develop more infectious complications, hypothesizing a weaker immune system with a potential impairment of leukocyte function. However, in this study, only a slight increase in superficial wound infections has been observed (13% vs. 6%), whereas no differences were found for intra-abdominal abscesses, which have a greater impact on the postoperative course.

One of the reasons not to postpone surgery in jaundiced patients with resectable and borderline resectable cancer, is the fear to increase surgical complications due to the need of a biliary stent (8). Actually, in this study the percentage of preoperative biliary drainage was the same in both groups, highlighting that patients referring to a tertiary center, rarely can be candidate to immediate surgery. Similar to surgical morbidity, complications affecting cardio-respiratory or renal systems were comparable in the two groups, indicating that a prolonged combination chemotherapy did not predispose to medical complications after surgery. Previous studies suggested a protective effect of neoadjuvant treatment on pancreatic fistula (28–30), hypothesizing a change in pancreatic parenchyma structure induced by chemoradiotherapy resulting in increased fibrosis and pancreatic duct dilation. In the present analysis, before propensity score adjustment there was a close-to-significant reduction of the overall pancreatic leak rate in the preoperative therapy group. After propensity score adjustment including Callery's fistula risk score, this slight difference was lost.

An increase of about 30 min of the duration of surgery was detected in the NACT group, both before and after the adjustment; this finding was expected, because peripancreatic tissues are affected by edema and fibrosis after preoperative therapy, increasing the complexity of operation, even in the absence of vascular infiltration. However, there was no greater blood loss or transfusion need increase; in this context, an interesting finding was that chemotherapy did not result in a reduction of preoperative hemoglobin in the treated group.

In a subgroup analysis including only NACT patients, postoperative outcomes were similar between patients who received gemcitabine-based combination chemotherapy vs. mFolfirinox. This confirms recent findings from a multicenter retrospective study comparing survival outcomes in patients treated with mFolfirinox and gemcitabine/nab-paclitaxel (1). Larger prospective studies comparing the two regimens are needed to evaluate more comprehensive outcomes including treatment toxicity during NACT, which may even delay or prevent indication to surgery.

### Strengths and Limitations

As other previous studies on this topic, the present one has several limitations. First of all, its retrospective nature that carries intrinsic patient selection biases, missing information and potential inaccuracy in collected data. Second, the sample size of our cohort is relatively small, which may increase the risk of type II error and false negative results especially for less common outcomes such as major morbidity and SSIs. However, the use of the CCI as primary outcome provides a sensitive endpoint even with smaller sample sizes (27). In addition, an important limitation was that a significant proportion of patients underwent preoperative treatment in other hospitals, so they received different chemotherapy regimens with variable duration; further, radiotherapy was sometimes associated to chemotherapy, although in a minority of cases (12.6%). Another shortcoming in this study was the absence of preoperative nutritional status data and patient reported outcomes, which may have provided greater information on the effect of chemotherapy on patient overall condition and quality of life. Finally, <10% of patients included in this analysis had resectable disease at diagnosis, which may limit the generalizability of our findings to that subgroup of patients.

Nevertheless, the present study has significant strengths: it is a single center series including a large cohort of preoperatively treated patients in a short time period with high profile chemotherapeutic schedules. We chose to include only patients who underwent PD to highlight the impact of preoperative treatment on the highest-risk operation. Furthermore, to limit the influence on postoperative outcomes of known risk factors other than preoperative chemotherapy, the comparison between the two cohorts was based on a propensity score match employing patient-specific and procedural variables. In addition, the main outcomes analyzed included only validated and reproducible measures, such as the CCI, CDC-defined SSIs, and post-pancreatectomy complications according to ISGPS definitions.

## Conclusions

The present study suggests that modern combination chemotherapy with proven activity against pancreatic cancer, can be safely used preoperatively, without affecting morbidity and postoperative recovery. Future studies should evaluate outcomes for neoadjuvant combination chemotherapy in areas that have been marginally considered to date, as for patients with resectable pancreatic cancer.

## Data Availability Statement

The datasets generated for this study are available on request to the corresponding author.

## Ethics Statement

Ethical review and approval was not required for the study on human participants in accordance with the local legislation and institutional requirements. Written informed consent for participation was not required for this study in accordance with the national legislation and the institutional requirements. We stated that the review board waived the need for ethics approval due to retrospective nature of the study.

## Author Contributions

NP and GBa designed the study. MP, LC, GBe, and FD collected and entered data. NP and FD analyzed the data. NP and GBa wrote the manuscript. NP, SP, SC, DT, RC, MR, MF, and GBa reviewed the manuscript. All authors read and approved the final version of the manuscript.

### Conflict of Interest

The authors declare that the research was conducted in the absence of any commercial or financial relationships that could be construed as a potential conflict of interest.
